# BiU-Net: A Biologically Informed U-Net for Genotype Imputation

**DOI:** 10.21203/rs.3.rs-6797863/v1

**Published:** 2025-08-26

**Authors:** Lei Huang, Kuan-Jui Su, Meng Song, Chuan Qiu, Loren Gragert, Jeffrey Deng, Zhe Luo, Qing Tian, Ping Gong, Hui Shen, Chaoyang Zhang, Hong-Wen Deng

**Affiliations:** University of Southern Mississippi; Tulane University; Xi’an Shiyou University; Tulane University; Tulane University; Dartmouth College; Tulane University; Tulane University; U.S. Army Engineer Research and Development Center; Tulane University; University of Southern Mississippi; Tulane University

**Keywords:** genotype, imputation, U-Net, deep learning

## Abstract

Missing genotypes reduce statistical power and hinder genome-wide association studies. While reference-based methods are popular, they struggle in complex regions and under population mismatch. Existing reference-free deep learning models show promise in addressing this issue but often fail to impute rare variants in small datasets. We propose BiU-Net, a biologically informed U-Net model that segments genotype data and encodes positional information to preserve the genomic context. Evaluated on the 1000 Genomes Project, Louisiana Osteoporosis Study, and Simons Genome Diversity Project datasets, BiU-Net outperformed Beagle and sparse convolutional denoising autoencoder in overall metrics and in metrics stratified by minor allele frequency.

## 1 Background

Missing genotype data reduces the statistical power of genome-wide association studies (GWASs) and compromises the detection of genotype–phenotype associations [1, 2]. Genotype imputation addresses this issue, but the accuracy—particularly for rare variants—depends heavily on the size and ancestral match of the reference panel [3, 4].

While classic reference-based methods such as Beagle [5], Minimac [6], and Impute5 [7] perform well for common variants, their performance declines for rare variants, especially when the population structure differs between the reference and target cohorts. Moreover, rare variant detection is inherently difficult because of weak linkage disequilibrium (LD) and sequencing coverage requirements[8], and filtering of low-confidence loci during postprocessing often exacerbates this problem[9, 10].

Deep learning-based, reference-free methods offer an alternative by learning directly from complete target haplotypes. Convolutional neural networks (CNNs) have been widely adopted because of their effectiveness in modeling structured inputs, such as in the sparse convolutional denoising autoencoder (SCDA) model [11, 12]. However, CNNs face limitations when applied to very long genotype sequences. Capturing long-range dependencies necessitates deeper architectures, which substantially increase model complexity. This, in turn, heightens the risk of overfitting dominant signals when training data are limited, ultimately impairing the imputation of rare variants.

To circumvent this, prior works have split genotype sequences into smaller blocks and trained separate models per region [13–15]. While this strategy is effective for capturing local patterns, it introduces fragmentation, increasing model complexity and impairing global consistency. A key reason behind this design choice might stem from the prevalent treatment of genotype data as tabular—where each row represents an individual and each column corresponds to a locus—implicitly assuming that loci are independent features. Under this view, stacking blocks of genotypes into training a unified model becomes infeasible, as there is no inherent notion of order or continuity. However, this assumption fails to capture the biological reality that genotypes are derived from linear DNA sequences and exhibit sequential structures. Traditional HMM-based methods, such as Beagle and IMPUTE, inherently model this sequential dependency, allowing for more coherent imputation across genomic regions. Disregarding this structure limits the ability of deep learning models to generalize across blocks, especially for rare variants that depend on long-range haplotype context.

To address these challenges, we propose a biologically informed U-Net (BiU-Net) model trained on overlapping genotype segments with positional encoding to preserve the genomic context. Unlike prior models, BiU-Net uses a unified architecture over all segments, enabling consistent learning while benefiting from data augmentation through segmentation. A post inference reassembly process reconstructs the full sequence from overlapping predictions.

We benchmark BiU-Net against Beagle5.5 [16, 17]—a robust reference-based method—and SCDA, a widely adopted CNN-based model that has inspired many subsequent deep learning approaches, using a unified pipeline across three datasets: the 1000 Genomes Project (1KGP) [4], the Louisiana Osteoporosis Study (LOS) [18], and the Simons Genome Diversity Project (SGDP) [19]. We also evaluate how population structure influences imputation performance to assess the model’s robustness in diverse and underrepresented cohorts.

## 2 Results

We evaluated BiU-Net’s genotype imputation performance against Beagle and SCDA across three datasets—1KGP, LOS, and SGDP—under varying missingness rates and minor allele frequency (MAF) bins. All models were benchmarked using accuracy, R^2^, precision, recall, and F1 scores, with detailed metrics provided in Supplementary Tables S1–S20 and Figures S3–S10.

### 2.1 Imputation performance comparison across datasets

**Figure 1:**
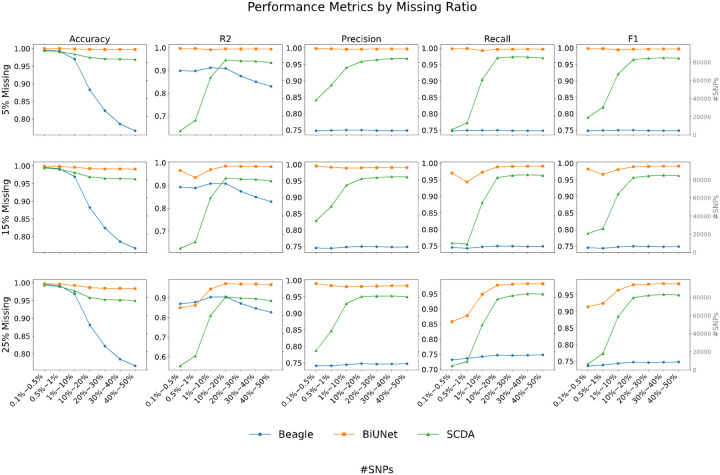
Performance comparison of BiU-Net, Beagle, and SCDA for genotype imputation on the 1KGP dataset, with each row representing a different missing data ratio (5%, 15%, and 25% missing), and each column showing different performance metrics (accuracy, R^2^, precision, recall, and F1 score). For each subplot, the x-axis shows MAF bins, whereas the y-axis displays the performance metric values. Line plots with different colors represent each model’s performance across MAF bins, with blue bars in the background indicating the number of SNPs in each bin (shown on the secondary y-axis). The centered text in each subplot shows the overall metric scores for each model.

On 1KGP chromosome 22, BiU-Net outperformed SCDA and Beagle across all metrics at low missingness (5%), particularly for rare variants (MAF < 1%). For example, in the 0.5–1% MAF bin, BiU-Net achieved R^2^ = 0.9965, whereas Beagle and SCDA achieved 0.8981 and 0.6813, respectively (Table S1). Even at higher levels (15%, 25%), BiU-Net maintained strong performance. Beagle slightly surpassed BiU-Net in the rarest MAF bin (< 0.5%) at 25% missingness, but BiU-Net consistently led in precision, recall, and F1 across MAF bins (Tables S1–S3, Fig. 1).

In the LOS dataset, BiU-Net achieved the highest overall R^2^ across all missingness levels: 0.9932 (5%, Table S4), 0.9787 (15%, Table S5), and 0.9634 (25%, Table S6); it also ranked first across both the rarest (≤ 0.5%) and most common (≥ 40%) MAF bins (Tables S4–S6). Confusion matrix analysis (Figure S3) revealed that BiU-Net outperformed Beagle in phased genotype recovery, especially at heterozygous sites.

In the SGDP dataset, which includes more complex population structure, BiU-Net remained effective even in the HLA region. It consistently achieved R^2^ >0.97 for SNPs with MAF ≤ 0.5% across all missingness levels (Tables S10–S12), whereas SCDA and Beagle struggled with rare variants and missing SNPs. Beagle also excluded 4.69–20% of the SNPs across various MAF bins lacking reference matches (Table S19–20). We found that both BiU-Net and SCDA exhibited consistent performance whether benchmarked on the SNPs retained by Beagle or on the full set of SNPs, confirming the robustness of reference-free models. Beagle’s performance is reported on its retained subset of SNPs, while BiU-Net and SCDA are evaluated on the complete SNP sets. To avoid redundancy and conserve space, we report the results of all three models in Tables S7–S12 using this setup.

### 2.2 Cohort-Specific Performance

In single-cohort experiments on the LOS dataset, BiU-Net showed robust generalizability and rare variant recovery. Beagle maintained stable R^2^ values across cohorts (e.g., ~ 0.91 at 5% ~ 25% missing levels on both African American and Caucasian samples), suggesting its reliance on the reference panel rather than the cohort structure (Tables S13–S18). SCDA, however, significantly decreased in performance for the African American cohort, with R^2^ values falling from 0.9881 (Table S4) to 0.8730 (Table S13) at 5% missingness, and down to 0.6713 at 25% missingness (Table S15).

BiU-Net improved slightly in the Caucasian cohort, achieving overall R^2^ = 0.9962 at 5% missingness (Table S16), compared with 0.9932 in the admixed group. This likely reflects benefits from population homogeneity and longer LD blocks. In the African American cohort, BiU-Net showed minor decreases in overall R^2^ (e.g., 0.9276 at 25% missingness Table S15) but still outperformed SCDA and Beagle, especially in the rarest variants (R^2^ = 0.9976, 0.9587, 0.9009 for MAF < = 0.5% at 5%, 15%, and 25% missingness, respectively; Table S13–S15).

**Figure 2:**
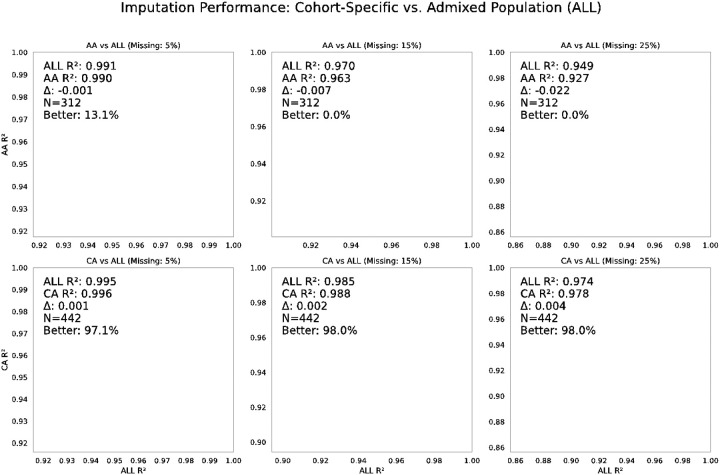
Comparison of imputation performance (R^2^) between cohort-specific models and a model trained on the admixed population (ALL) across varying missing data rates (5%, 15%, and 25%). Each scatter plot compares the per-sample imputation performance (R^2^) of the BiU-Net model trained on a specific cohort (African American [AA] or Caucasian [CA]) versus the model trained on the full admixed population (ALL). Points below the diagonal indicate samples where the ALL-trained model outperformed the cohort-specific model. In the African American cohort (top row), the ALL model consistently outperformed the AA-specific model across all missing data rates, suggesting improved generalizability due to the inclusion of broader genetic diversity in training. In contrast, for the Caucasian cohort (bottom row), the performance differences between the CA-specific and ALL models were minimal, with the CA-specific model slightly outperforming the ALL model in some cases.

To further support the cohort-specific trends observed in the per-SNP benchmarking results, Fig. 2 presents a per-sample comparison between BiU-Net models trained on cohort-specific versus admixed data. This individual-level perspective complements the previous SNP-level analysis by illustrating how model performance varies across samples, revealing consistent advantages of the admixed model for genetically diverse populations and minimal gains from cohort-specific training in more homogeneous cohorts.

For African American individuals, BiU-Net models trained on the admixed cohort consistently outperformed those trained on African American samples alone, with average per-sample R^2^ gains increasing as the missingness level rose. In contrast, for Caucasian individuals, the model trained exclusively on Caucasian samples achieved slightly higher performance than the admixed model when evaluated on the same cohort, although the differences were minimal. These findings suggest that incorporating diverse training data improves generalizability for genetically complex populations, whereas more homogeneous populations may benefit modestly from cohort-specific training.

### 2.3 Imputation performance on rare variants

The improvements of BiU-Net were especially notable for the rarest variants. For example, in the LOS CA cohort with 5% missing data, BiU-Net achieved R^2^ = 0.9970 for SNPs with MAF ≤ 0.5%, whereas SCDA had an R^2^ of 0.3219 (Table S16). In the AA cohort at the same missing data level, BiU-Net scored R^2^ = 0.9976, whereas SCDA scored 0.5640 (Table S13). In the SGDP HLA region under 25% missingness, BiU-Net maintained R^2^ = 0.9830, whereas SCDA decreased to 0.1792 (Table S12). These gains are attributed to BiU-Net’s segmentation strategy, U-Net-based architecture, positional encoding, and hybrid loss, all of which enhance rare variant sensitivity and generalizability.

## 3 Discussion

BiU-Net addresses critical limitations of deep learning-based genotype imputation, particularly for rare variant recovery, population generalization, and scalability without reliance on reference panels. Its segmentation strategy enables the model to effectively process long genotype sequences by breaking them into shorter, overlapping windows—reducing input dimensionality while preserving local haplotype structure. This not only improves training efficiency but also acts as a form of data augmentation, especially valuable for datasets with limited sample sizes, such as SGDP.

Unlike SCDA, which uses full-length sequences and struggles with rare variants in complex populations, BiU-Net resolves the positional ambiguity introduced by segmentation through the integration of normalized SNP coordinates. This allows the model to disambiguate structurally similar yet genomically distant segments, thereby improving prediction accuracy in both common and rare variant contexts.

BiU-Net’s U-Net-based architecture leverages skip connections for deep representation learning while maintaining a relatively low parameter count (9.7 million vs. SCDA’s 17 million), which accelerates convergence and reduces the risk of overfitting. Additionally, the proposed hybrid loss function jointly optimizes phased genotype classification and dosage prediction, resulting in balanced performance across multiple evaluation metrics. The model’s effectiveness in both homogeneous (e.g., Caucasian LOS cohort) and genetically diverse or admixed cohorts (e.g., African American LOS and SGDP samples) demonstrates its robustness across varying population structures.

Nonetheless, performance in the African American cohort under high missingness levels (e.g., 25% in Table S15) showed slight underperformance in low-frequency variants, indicating that additional tuning—such as adjusting learning rate schedules, segment size, or training curricula—may further improve generalizability in complex populations. These observations also highlight the importance of incorporating diverse training cohorts to avoid population-specific performance degradation.

Importantly, the segmentation framework developed for BiU-Net is model-agnostic and can be adapted to other neural architectures, including transformers and graph-based models. This provides a foundation for scalable, reference-free genotype imputation frameworks that can extend beyond current limitations.

Future directions include applying BiU-Net to whole-genome data, integrating population-level priors, and exploring transfer learning approaches for rare populations. Expanding benchmarking across broader ancestral groups will also be essential for ensuring equitable imputation accuracy in global genomic studies.

## 4 Conclusion

This study presents BiU-Net, a biologically informed U-Net model that enables reference-free genotype imputation with high accuracy across diverse populations and rare variant contexts. Through a combination of segmentation-based training, positional encoding, and hybrid loss optimization, BiU-Net achieves superior performance over both traditional and deep learning baselines. Importantly, the segmentation framework is architecture-agnostic and extensible, supporting broader application in future reference-free imputation studies. These findings demonstrate the potential for deep learning models to improve equity and resolution in population genomics.

## 5 Methods

This section outlines the data sources, preprocessing procedures, and model architecture employed in this study. We first describe the datasets and associated quality control steps, followed by the genotype segmentation strategy developed to facilitate model training. Finally, we present the design and implementation of the BiU-Net model and the training details, including the proposed hybrid loss function that jointly optimizes phased genotype classification and dosage prediction to improve training convergence and rare variant sensitivity.

### 5.1. Dataset

To demonstrate the consistency of the effectiveness of our model, we utilize three datasets:

#### 1000 Genomes Project (1KGP) dataset.

The 1KGP dataset is renowned for capturing a broad spectrum of human genetic diversity, with samples drawn from multiple global ancestries such as South Asian, East Asian, African, Admixed American, and European populations. Its comprehensive coverage and diverse sampling make it a valuable/common resource for detailed genetic analyses and robust benchmarking in genomic studies. In this study, we utilize this dataset as the reference panel for Beagle when imputing other datasets.

#### Louisiana Osteoporosis Study (LOS) dataset.

**Figure 3:**
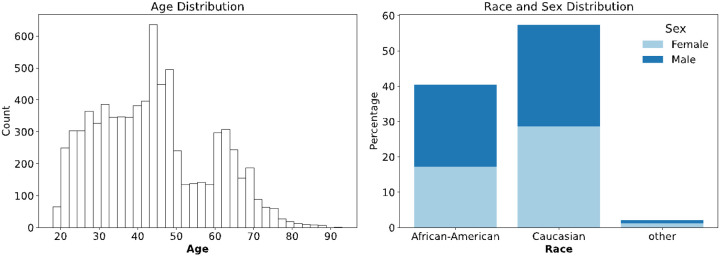
Demographic characteristics of the subjects in the Louisiana Osteoporosis Study (LOS) dataset. Left panel: Age distribution of the study participants, with frequency counts across different age groups. Right panel: Distribution of participants by race and sex, displaying the number of subjects categorized into African American, Caucasian, and other racial groups, with further stratification by sex (dark blue for male, light blue for female).

In addition to the 1KGP dataset, we included our in-house LOS dataset. The details of the whole genome sequence data were described in our previous study[18]. The overall statistics of this dataset are illustrated in Fig. 3.

We excluded all racial groups except for Caucasians and African Americans, as the sample sizes for other races were insufficient for effective model training. After this exclusion, the LOS dataset contains 7,504 samples. Compared to the 1KGP dataset, the LOS dataset contains fewer SNPs but comprises significantly more samples.

#### Simons Genome Diversity Project (SGDP) dataset.

The Simons Genome Diversity Project offers one of the most comprehensive and globally diverse collections of high-quality human genome sequences to date. Unlike earlier initiatives such as the 1000 Genomes Project, SGDP employed high-coverage sequencing (> 30×) and a specialized genotyping pipeline tailored for population genetic studies. Our study utilizes the 278 publicly available SGDP genomes, spanning 127 distinct populations across seven geographic regions: African (44), Native American (22), Central Asian/Siberian (27), East Asian (46), Oceanian (25), South Asian (39), and West Eurasian (75) populations. Owing to the limited sample sizes per individual population, we evaluate imputation performance across the entire SGDP dataset rather than conducting region-specific training. This strategy provides a more robust and stable evaluation, ensuring that models benefit from the full range of genetic diversity.

### 5.2. Data preprocessing

The preprocessing pipeline consisted of two stages: a quality control (QC) phase and genotype segmentation.

#### Quality Control.

The QC process began by generating reference panels from the 1000 Genomes Project (1KGP) dataset, which is already phased and aligned with the GRCh38 genome build. The QC steps included removing duplicated variants and retaining only biallelic SNPs. For chromosome 22, this resulted in 2,548 samples and 993,880 SNPs, which were subsequently used as the reference panel for Beagle-based imputation.

The SGDP dataset was lifted over from GRCh37 to GRCh38 via Picard [20], whereas phasing of the LOS dataset was performed via Beagle. Before the QC, all the samples were phased and aligned to the GRCh38 reference genome to ensure consistency.

QC was conducted following a pipeline similar to that described by Marees et al [21], utilizing tools such as BCFtools [22–27] and PLINK [28]. Stratification steps were additionally incorporated to ensure that a uniform set of SNPs was retained across subsets within each cohort. The full pipeline is detailed below (the 1KGP dataset had already undergone the first three QC steps during reference panel preparation):

1. Retain only biallelic SNPs.
2. Exclude individuals with >5% missing genotypes.
3. Remove SNPs with missing genotypes.
4. Apply an 8:1:1 train-validation-test split, stratified by cohort (populations or regions) and sex. For the LOS dataset, samples were first categorized by cohort and sex, then randomly split, and finally aggregated by cohort to prevent data leakage when tested across cohorts.
5. Apply Hardy-Weinberg Equilibrium (HWE) and MAF thresholds: HWE > 1×10^−6^ and MAF ≥ 0.1% on each split independently.
6. Retain only overlapping SNPs across all subsets of the same cohort to ensure consistency in model training and testing.

All datasets were obtained from publicly available repositories. Comprehensive details regarding data acquisition and preprocessing are provided in the accompanying GitHub repository. Table 1 summarizes the dataset statistics after QC for each cohort.

Summary statistics of each dataset following preprocessing and training–validation–test splitting. The datasets include chromosome 22 from the 1000 Genomes Project (1KGP), chromosomes 22 and 6 (HLA region) from the Simons Genome Diversity Project (SGDP), and chromosome 22 from the Louisiana Osteoporosis Study (LOS). The LOS dataset is further stratified into three cohorts: ALL (combined African American and Caucasian samples), AA (African American cohort), and CA (Caucasian cohort). For each dataset and cohort, we report the number of samples in the training, validation, and test sets, along with the total sample count and the number of SNPs retained after quality control. The training-validation-test split follows an 8:1:1 ratio while maintaining cohort representation. To ensure a fair comparison between the ALL-population model and cohort-specific models, no samples in the AA or CA test sets were included in the training or validation sets of the ALL cohort, thereby preventing any potential data leakage.

#### Segmentation.

**Figure 4:**
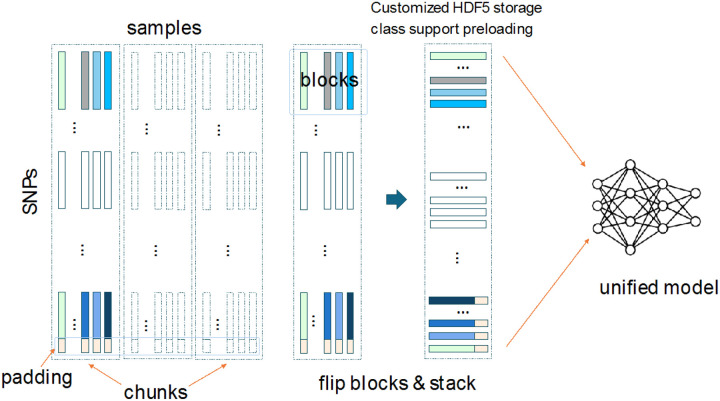
Segmentation of an existing genotype dataset: The genotype matrix extracted from the VCF format undergoes a series of preprocessing steps. First, it is encoded and padded and then split by samples into smaller chunks. Each chunk is further divided into overlapping segments (blocks), which are flipped and stacked. These processed data are then shuffled and segmented, making them ready for training. A unified model is subsequently trained on the resulting data in batches.

To facilitate deep learning on large genomic datasets, we introduced a segmentation strategy that partitions phased genotypes into shorter, overlapping blocks. This reduces the sequence length, improves the memory efficiency, and helps capture localized patterns while easing model generalizability. Genotypes were first encoded to preserve phasing: missing as 0, padding as 5, homozygous reference (A∣A) as 1, heterozygous (A|a, a|A) as 2 and 3, and homozygous alternate (a|a) as 4. The samples were chunked for memory efficiency and then segmented into fixed length, overlapping windows. The segments were padded and transposed (SNPs as columns) before being stored in an HDF5 format, which supported both full and batchwise loading. A detailed description of this method is illustrated in Fig. 4.

We evaluated segment lengths (64–512 SNPs, with overlap of 4 SNPs) on chromosome 22 of the 1KGP European cohort and found that 128 SNPs provided the best trade-off between training diversity and model convergence (Supplementary Figures S1, S2). While the size of the overlap had minimal impact on performance in large, well-powered datasets, increasing overlap improved training stability and imputation accuracy in more challenging settings—particularly in small cohorts, admixed populations, and structurally complex regions. Therefore, we used 128-SNP segments with a 16-SNP overlap for most experiments but increased the overlap to 64 SNPs for the HLA region of chromosome 6 in the SGDP dataset to enhance model performance under these difficult conditions.

At inference, the same segmentation settings are applied, with an added reassembly step. Overlaps are resolved by selecting the highest Softmax logits across segments. Sample identifiers and SNP segments orders are preserved throughout, ensuring accurate reconstruction in a desegmentation process.

### 5.3. Model

The SCDA model used in our experiment is based on the original study [11]. The encoder processes fixed-length genotype segments through multiple convolutional blocks, each with a 1D convolution, ReLU activation, max-pooling, and dropout. As depth increases, the number of channels doubles, enabling the model to capture complex patterns, whereas max-pooling reduces sequence length for efficiency. The decoder mirrors this structure in reverse, halving channels and using upsampling to reconstruct genotypes. We extended the original SCDA to six layers to accommodate the greater complexity of our dataset.

The proposed BiU-Net builds on SCDA with several key improvements. It adopts a U-Net [29] structure with skip connections, facilitating deeper feature reuse and faster convergence. Despite its depth, BiU-Net is more efficient, using 9.7 million parameters versus SCDA’s 17 million, without sacrificing accuracy. Unlike previous models, BiU-Net is trained as a unified model across all genotype segments. Owing to sparse variation, many segments share identical or near-identical patterns—a problem that is compounded by the fact that genotype data are sequential and biologically contextualized; identical patterns from different genomic regions may have distinct functional implications. Without introducing any mechanism to integrate the positional information of where each segment originates, the model can become confused during training.

To resolve this positional ambiguity, we add a min-max normalized positional channel per segment, which is appended alongside the one-hot encoded genotypes. This positional input acts as a biological “watermark,” enabling the model to distinguish between structurally similar but genomically distinct segments. We refer to this approach as biologically informed modeling, which inspired the name BiU-Net.

**Figure 5:**
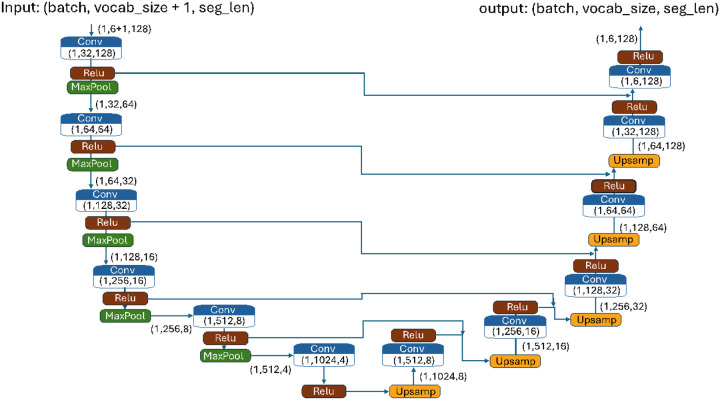
The architecture of the proposed biologically informed U-Net (BiU-Net) model for genotype imputation. The model adopts a U-Net structure and is trained via a denoising approach. The input data have dimensions (batch_size, vocabulary_size + 1, segment_length), and the additional dimension to the vocabulary size incorporates normalized genomic positions as “hints” or “watermarks.” These positional cues enable the model to distinguish between identical SNP sequences from different genomic regions in the original genotype dataset. The architecture processes genetic data through 1D CNN layers (blue), ReLU activations (brown), and max-pooling operations (green) in the encoder. Skip connections preserve information between the corresponding encoder-decoder levels. Each block displays the output tensor dimensions, with the model progressively squeezing spatial dimensions while increasing feature depth in the encoder path, followed by a reverse process in the decoder path. These skip connections facilitate information retention, accelerate training convergence, and significantly reduce the number of model parameters compared with the SCDA model.

This segmentation-based training strategy also expands usable training data, making it feasible to train BiU-Net as a reference-free model on datasets with few samples but many variants, such as SGDP. The full architecture is illustrated in Fig. 5.

### 5.4. Model training

To address the challenge of class imbalance in the dataset, particularly for rare variants, we employed a hybrid approach that combines both focal loss [30] and dosage regression during training.

To be more specific, suppose that we have N genotypes in a batch, the ground truth is $y_{i}$ and the predicted genotype is ${\overset{ˆ}{y}}_{i}$, we can express the total loss as the weighted sum of the classification loss and the dosage prediction loss:

(1) $$ℒ=(1-\lambda ){ℒ}_{\text{classification}}+\lambda \;{ℒ}_{\text{dosage}}$$

where the classification loss ${ℒ}_{\text{classification}}$ can be calculated as:

(2) $${ℒ}_{\text{classification}}=\frac{1}{N}\sum_{i=1}^{N}\setminus \text{varvec}\;\text{P}\left(y_{i},{\overset{ˆ}{y}}_{i}\right){\left(1-p_{i}\right)}^{\gamma }{\;ℒ}_{CE}\left(y_{i},{\overset{ˆ}{y}}_{i}\right)$$

$p_{i}$ is the probability of the correct label $y_{i}$ being predicted, which can be calculated from the model’s logit z by applying the Softmax function:

(3) $$p_{i}\equiv p_{\text{index}\left(y_{i}\right)=k}=\frac{\text{exp}\left(z_{k}\right)}{\sum_{\;j}\text{exp}\;\left(z_{j}\right)}$$

k and j represent indices of the genotype encoding dimension. ${ℒ}_{CE}\left(y_{i},{\overset{ˆ}{y}}_{i}\right)$ is the cross-entropy loss which can be calculated as:

(4) $${ℒ}_{CE}\left(y_{i},{\overset{ˆ}{y}}_{i}\right)=-\text{log}p_{i}$$

${\left(1-p_{i}\right)}^{\gamma }$ is the focal weighting term with hyperparameter γ, as in the vanilla focal loss equation. The context-aware penalty term $\setminus \text{varvec}\;\text{P}\;\left(y_{i},{\overset{ˆ}{y}}_{i}\right)$ which is different from the explicit class imbalance weighting factor $\alpha_{y_{i}}$ in the original focal loss paper. Compared with a normalized $\alpha_{y_{i}}$ which is used to give different emphases for imbalanced classes, the penalty $\setminus \text{varvec}\;\text{P}\;\left(y_{i},{\overset{ˆ}{y}}_{i}\right)$ acts like the reward in the reinforcement learning theory, although it works inversely: To incorporate contextual information into the loss function, we define a penalty score matrix for dosage prediction, as shown in Table 2.

In this penalty score matrix, each entry specifies a penalty $\setminus \text{varvec}\;\text{P}\left(y_{i},{\overset{ˆ}{y}}_{i}\right)$ on the basis of the relationship between the true genotype $y_{i}$ (rows) and the predicted genotype ${\overset{ˆ}{y}}_{i}$ (columns). The values were empirically assigned to reflect the biological impact of different prediction errors: higher penalties are given to incorrect predictions that are farther from the true dosage, whereas less critical penalties are applied in cases where the predicted dosage is correct, but the phasing is incorrect (e.g., mislabeling of heterozygous alleles).

The table presents the penalties under different contexts, with the penalty score $\setminus \text{varvec}\;\text{P}\;\left(y_{i},{\overset{ˆ}{y}}_{i}\right)$ used as the extra weighting term to the focal weighting term ${\left(1-p_{i}\right)}^{\gamma }$. Each entry $\setminus \text{varvec}\;\text{P}\;\left(y_{i},{\overset{ˆ}{y}}_{i}\right)$ defines the penalty applied when the true phased genotype $y_{i}$ (rows) is predicted as ${\overset{ˆ}{y}}_{i}$ (columns). The scores are empirically designed to reflect the biological relevance of prediction errors— larger penalties are assigned to incorrect predictions with greater dosage deviation, whereas smaller penalties are used for phase-switch errors (e.g., cases where heterozygous alleles are misphased, but the total dosage is still correct). This matrix acts as a context-aware weighting mechanism in the loss function, complementing the focal weighting term ${\left(1-p_{i}\right)}^{\gamma }$ and encouraging the model to prioritize biologically meaningful accuracy. Notably, the subscript i refers to the i-th SNP in the loss calculation, whereas the penalty score $\setminus \text{varvec}\;\text{P}\;\left(y_{i},{\overset{ˆ}{y}}_{i}\right)$ is based solely on the dosage-level mismatch and is independent of the SNP index i.

We arbitrarily set γ as 2.0, together with the predefined penalty scores throughout our experiments without fine-tuning them, as we achieved satisfactory imputation performance compared to cross-entropy or vanilla focal loss.

The dosage prediction loss ${ℒ}_{\text{dosage}}$ is defined as the MSE loss of the predicted dosage against the ground truth dosage:

$${ℒ}_{\text{dosage}}=\frac{1}{N}\sum_{i=1}^{N}\left({\left|d_{i}-{\overset{ˆ}{d}}_{i}\right|}^{2}\right)$$

The true dosage $d_{i}$ is assigned as follows:

(6) $$d_{i}=\left\{\begin{array}{ll} 0 & \text{if}\;y_{i}\;\text{is}\;\text{a}\;\text{homozygous}\;\text{ref}\\ 1 & \text{if}\;y_{i}\;\text{is}\;\text{a}\;\text{heterozygous}\;\text{alternate}\\ 2 & \text{if}\;y_{i}\;\text{is}\;\text{a}\;\text{homozygous}\;\text{alternate} \end{array}\right.$$

According to the previously mentioned encoding scheme, the predicted dosage ${\overset{ˆ}{d}}_{i}$ can be computed from softmax probabilities as:

$${\overset{ˆ}{d}}_{i}=p_{k=2}+p_{k=3}+2*p_{k=4}$$

Finally, we use λ to control the contributions of the genotype classification loss and dosage prediction loss, so the overall loss can be represented as:

(8) $$ℒ=(1-\lambda )\frac{1}{N}\sum_{i=1}^{N}\setminus \text{varvec}\;\text{P}\;\left(y_{i},{\overset{ˆ}{y}}_{i}\right){\left(1-p_{i}\right)}^{\gamma }{ℒ}_{CE}\left(y_{i},{\overset{ˆ}{y}}_{i}\right)+\lambda \frac{1}{N}\sum_{i=1}^{N}\left({\left|d_{i}-{\overset{ˆ}{d}}_{i}\right|}^{2}\right)$$

When λ=1.0, the loss function is focused entirely on dosage prediction, whereas lower values of λ increase the weight of the classification-based focal loss. We set λ to 0.5 to balance the contributions of both loss terms. We found that the hybrid loss function achieved better performance than pure focal loss in our experiments.

To simulate realistic data sparsity during training, missing genotypes were dynamically masked within each batch. This approach prevented the model from overfitting to fixed patterns. We adopted curriculum learning by initially randomly masking 5% of the input to promote stable early convergence, then gradually increasing the missingness level up to 25% to improve robustness. For evaluation, test sets were randomly masked at fixed levels (5%, 15%, 25%) with three independent replicates per level, and average performance was reported to mitigate randomness. The accuracy, R^2^, precision, recall, and F1 score were used as evaluation metrics. For each MAF bin, metrics were computed by concatenating all SNPs rather than averaging per SNP within bins, preserving metric definitions and enhancing interpretability.

Part of the data preprocessing is conducted on the Cypress HPC cluster of Tulane University. All the models were trained on the high-performance computing (HPC) cluster of the Louisiana Optical Network Infrastructure (LONI) using PyTorch’s Distributed Data Parallel framework [31] with mixed-precision training. Owing to dynamic resource availability, experiments were conducted across CPU and GPU nodes. The largest GPU configuration used 12 nodes with 24 NVIDIA A100 80 GB GPUs. For example, training BiU-Net on chromosome 22 of the 1KGP dataset with a segmentation length of 128 and overlap of 16 generated 4,381,355 segments. For the full GPU setup, each epoch required approximately 14s, with convergence achieved in ~ 160 epochs. In contrast, SCDA, trained on full-length genotypes and limited sample sizes, required approximately 600 epochs to converge, despite its shorter per-epoch runtime.

## Supplementary Material

Supplementary Files

This is a list of supplementary files associated with this preprint. Click to download.

- 02SupplementarymaterialsforpaperBiUNetaBiologicalinformedUNetforGenotypeImputationsubmitBMCGenomeBiology.docx

## Figures and Tables

**Table 1:** Summary of the genotype datasets used in this study

Dataset	1KGP / Chr22	SGDP / Chr22	SGDP / Chr6 (HLA)	LOS: Chr22
**Cohort**	ALL	ALL	ALL	ALL/ AA/ CA
**#train**	2,035	213	213	6,001/ 2,479/ 3,522
**#val**	250	21	21	749/ 309/ 440
**#test**	263	44	44	754/ 312/442
**#total**	2,548	278	278	7504/ 3,100/ 4,404
**#SNPs**	241,113	117,318	47,740	257,352/ 307,599/ 188,399

**Table 2:** Score matrix for penalizing dosage prediction errors

Phased genotype		Predicted			
		1	2	3	4
**True**	**1**	0	3.0	3.0	3.0
	**2**	3.0	0	1.0	3.0
	**3**	3.0	1.0	0	3.0
	**4**	3.0	3.0	3.0	0

## Data Availability

The 1000 Genomes Project (1KGP) and Simons Genome Diversity Project (SGDP) datasets used in this study are publicly available through these URLs: 1KGP: https://hgdownload.soe.ucsc.edu/gbdb/hg38/1000Genomes/ https://ftp-trace.ncbi.nih.gov/1000genomes/ftp/release/20130502/ SGDP: https://sharehost.hms.harvard.edu/genetics/reich_lab/sgdp/ https://ftp.ensembl.org/pub/assembly_mapping/homo_sapiens/ https://ftp.ensembl.org/pub/release-110/fasta/homo_sapiens/dna/ All scripts and preprocessing methods used to retrieve, format, and prepare these datasets for analysis are available at our GitHub repositories: https://github.com/learnslowly/data The model and its training/testing/benchmarking code is located at: https://github.com/learnslowly/BiU-Net The scripts for Beagle’s imputation are located at: https://github.com/learnslowly/beagle The datasets from LOS that support this study’s findings are available from the principal investigator (H.W.D., hdeng2@tulane.edu) upon reasonable request. Access will be granted for academic research purposes, subject to IRB approval and the completion of a data use agreement. Additionally, the LOS WGS data is in the process of being deposited in the **AgingResearchBiobank** (https://agingresearchbiobank.nia.nih.gov/), where it will be made available to qualified researchers upon application and approval.
